# Medical Societies

**Published:** 1854-09

**Authors:** 


*Medical Societies.*

KNOX CO. MED. SOCIETY July 1st.—The regular annual meeting of the Knox Co. Medical Society was held at Galesburg, and the following officers were chosen for the ensuing year.

Dr. Jason Duncan, President; Dr. S. C. Patterson, Vice President; Dr. E. A Hamilton, Secretary; Drs. Cooper, Baily and Spaulding, Censors

Dr. J. W. Spaulding delivered before the society, a very able valedictory upon retiring from the office of presidency for the preceding year.

On motion it was voted that the address of Dr. Spaulding be sent to the Editors of the N. W. Medical and Snrgical Journal for publication.

The society then adjourned to meet in Knoxville, on the first Saturday in Oct. DR. J. DUNCAN, Pres.

DR. E. A. HAMILTON, Sec.

---

ANNUAL MEETING OF THE STARK CO. MED. SOCIETY, held in Wethersfield, Henry County, Ill., July, 13th 1854.

The President and Vice President being absent, on motion, Dr. Pinny was called to the chair.

Members present. Drs. Tho's. Hall, of Toulon, H. Nance, of Lafayette, A. A. Dunn, of Cambridge, Wm. Chamberlain, of Toulon, E. R. Boardman, of Elmira, G. I. Samper, of Oceola, Millikan, of Wyoming, A. R. Bodley, of Victoria, E. Pinny, G. P. Bancroft, and T. D. Fitch, of Wethersfield.

On motion of Dr. Chamberlain, the minutes of the special meeting, held in Toulon May, 23rd, 1854, were accepted.

Dr. Lamper moved to expunge—lost.

Dr. Lord, of Annawan, was proposed by Dr. Milliken, and unanimously elected a member of this society.

Society then proceeded to the election of officers for the ensuing year, when the following members were eleeted. President: Dr. Tho's. Hall, Toulon, Stark Co. Ill. Vice President: Dr. E. R. Boardman, of Elmira, Stark Co. Ill. Secretary: Dr. T. D. Fitch, of Wethersfield, Henry Co., Ill.

The President appointed Drs. H. Nance, Wm. Chamberlain and Milliken, Censors.

On motion of Dr. Samper, the charges of unprofessional conduct preferred against Dr. Hall, by Dr's. Boardman and Samper, were taken up.

Dr. Chamberlain moved to lay on the table—lost

The merits of the question were then discussed by most of the members present.

Vote resulted as follows :—

Yeas—Drs. Nance, Bodley, Boardman, Fitch. Nays—Dr's. Hall, Chamberlain, Dunn, Milliken and Bancroft.

Dr. Pinny, President *pro tem.* giving casting vote in favor of Dr. Hall.

Drs. Samper, Nance, Boardman and Bodley withdrew from the society.

On motion of Dr. Chamberlain, the withdrawal of the foregoing members, was recognized.

On motion of Dr. Fitch, a committee of three was appointed to present a fee-bill, for the action of this society at its next regular meeting.

Drs. Fitch, Chamberlain and Pinny were appointed said committee.

On motion, certificates of membership and good moral character were granted to Drs. Lord and Harlan.

The President appointed Dr. R. F. Henry in place of Dr. Nance, Censor.

On motion, society adjourned to meet in Toulon on the 4th Tuesday in October next.

THO'S. HALL, M. D., Pres.
T. D. FITCH, M. D. Sec.

---

COOK CO. MEDICAL SOCIETY.—The regular annual meeting of this Society was held on Tuesday evening, in the office of DR. PARKER.

A respectable number of members were present, and the meetin was an interesting and profitable one. Dr. De Laskie Miller, of Chicago, read a paper on *cholera infantum*, and the use of Quinine in its treatment, The essay was listened to with much attention, and was followed by a discussion which occupied the remainder of the evening. Dr. Miller was requested to furnish a copy of his paper for publication in the Journal. Hence we shall not attempt to give any detailed account of it. D.

